# Untangling pathways from digital instructional leadership to AI pedagogy: a diffusion of innovation theory perspective

**DOI:** 10.1186/s40359-025-03792-9

**Published:** 2025-12-06

**Authors:** Chuan-Chung Hsieh, Sandrotua Bali, Hui-Chieh Li

**Affiliations:** 1 Department of Education and Learning Technology, National Tsing Hua University, No. 101, Section 2, Guangfu Rd, East District, Hsinchu City, 300 Taiwan, ROC; 2 Center for Teacher Education, National Tsing Hua University, No. 101, Section 2, Guangfu Rd, East District, Hsinchu City, 300 Taiwan, ROC; 3 Department of Business Administration, National Taipei University of Business, No.321, Sec. 1, Jinan Rd., Zhongzheng District, Taipei City, 100 Taiwan, ROC

**Keywords:** Digital instructional leadership, AI pedagogy, AI integration, Trust in AI, K–12 education, Diffusion of innovation theory

## Abstract

Drawing on Diffusion of Innovation Theory, this empirical study examines how digital instructional leadership impacts the integration of AI and AI pedagogy in K–12 schools. In addition, the study examines how trust in AI moderates the relationship between AI integration and AI pedagogy. To achieve this goal, a large-scale cross-sectional survey was administered to 842 K–12 teachers in Taiwan who had prior experience integrating AI tools into their classroom instruction. The data were analyzed using SPSS and Partial Least Squares Structural Equation Modeling (PLS-SEM). Results show that digital instructional leadership significantly predicts AI integration but has no direct effect on AI pedagogy. In addition, AI integration strongly predicts AI pedagogy and mediates the relationship between digital instructional leadership and AI pedagogy, whereas trust in AI does not moderate the relationship between AI integration and AI pedagogy. Both theoretical and practical contributions are discussed, offering insights for future research, policymakers, and teachers.

## Introduction

As AI becomes increasingly embedded in K–12 education, a key question arises: to what extent should teachers develop expertise in AI pedagogy for effective classroom practice? This question warrants deeper investigation, as AI is increasingly integrated in schools worldwide and is recognized for its potential to enhance teaching, learning, and school management [1]. Prior research has demonstrated that AI can facilitate personalized learning, support adaptive instructional pathways, and promote more inclusive learning environments [2, 3]. In addition to these pedagogical advantages, teachers can utilize AI tools to customize instructional content to meet students’ needs and handle routine administrative tasks more efficiently [4–6]. However, to fully realize these benefits, teachers need training to integrate these tools meaningfully into their instructional practice.

At the global policy level, UNESCO [7] highlights AI pedagogy as a core domain in the teacher competency framework. This framework is intended to guide teachers to (1) integrate AI tools effectively into curricula and classroom activities, (2) design learning experiences that use AI to personalize instruction, encourage critical thinking, and enhance digital literacy, and (3) address ethical issues while ensuring inclusive AI use in educational settings. Given AI’s growing presence in K–12 education, understanding AI pedagogy is essential, as it enables teachers to adopt innovative teaching approaches [8, 9]. In this vein, Chiu et al. [10] emphasize that teachers need to develop AI pedagogy in the current era, defined as the capacity to select and apply AI tools thoughtfully to enrich instruction and improve student learning. This reflects the growing trend of AI integration in K–12 education, where teachers are increasingly required to navigate regardless of their personal preferences.

Against this global backdrop, Taiwan is also taking the initiative. In late 2024, the Ministry of Education announced a program to fully integrate AI into elementary, junior high, and senior high school curricula [11]. The initiative emphasizes practical classroom applications, such as AI-assisted language learning tools and structured academic instruction. The focus is on developing core literacy, encompassing the knowledge, skills, and attitudes necessary to prepare students for both current and future challenges [12]. To achieve the goals, the government is promoting the use of generative AI learning tools and digital platforms to enhance student engagement and support personalized learning. For example, the Taiwan Adaptive Learning Platform (TALP), which initially launched in 2017 to strengthen technology-enhanced learning systems, has recently integrated generative AI to further enhance its capabilities. The upgraded platform now facilitates automated diagnostics and the identification of learning gaps to promote self-directed and adaptive learning [11].

However, the integration of AI in K–12 schools heavily depends on the leadership of school principals [13]. Pietsch and Mah [14] noted that principals need to cultivate digital instructional leadership (i.e., a proactive, digital mindset), as traditional instructional leadership alone is insufficient. While instructional leadership focuses on improving teaching and learning to enhance student outcomes [15], a more targeted form of digital instructional leadership is needed to support teachers in effectively integrating AI [14]. Additionally, principals’ attitudes and technological knowledge are factors that influence the integration of AI, enabling teachers to effectively apply it in the classroom [16]. This can be attributed to the role of digital instructional leadership, which may promote trust among teachers when incorporating AI for teaching purposes. However, when integrating AI in schools, trust may emerge as a factor that could weaken its use [17, 18]. For example, Foroughi et al. [17] emphasized that the moderating effect of trust in information provided by AI can weaken the impact of its usefulness on attitude to use it. Furthermore, unfavorable depictions of AI in the media, such as widespread job losses, could undermine confidence in AI technology, which possibly affects educators’ perceptions of its educational applications [18]. Consequently, trust in AI and its potential risks has become a widely discussed topic in the education sector.

Although a growing body of literature has examined the effects of AI integration in K–12 education [5, 16, 19], to the best of our knowledge, little attention has been paid to the critical role of digital instructional leadership. In particular, prior studies such as Pietsch and Mah [14] and Berkovich and Hassan [20] have conceptualized digital instructional leadership, yet they have not empirically examined how this form of leadership translates into teachers’ AI pedagogy. This gap is especially important because leadership influences teachers’ technology use largely through indirect, school-level mechanisms rather than policy direction alone. Moreover, while frameworks that emphasize AI pedagogy have been proposed [7], empirical investigations of AI pedagogy remain scarce [10]. Thus, the conceptual gap lies in understanding how digital instructional leadership shapes AI integration processes that ultimately lead to AI-integrated pedagogical practice, a relationship that has not yet been empirically examined.

To address this gap, the present study examines how digital instructional leadership influences AI integration and AI pedagogy among K–12 teachers. Grounded in the Diffusion of Innovation Theory, our study contributes to the ongoing discourse on how school leaders can guide AI adoption to support meaningful pedagogical transformation. Additionally, we investigate the potential moderating effect of trust in AI on the relationship between AI integration and AI pedagogy. The research questions guiding this study are:

- RQ1: Does principals’ digital instructional leadership influence AI integration and AI pedagogy among K–12 teachers?
- RQ2: Does teachers’ trust in AI moderate the relationship between AI integration and AI pedagogy?

This study is significant for its theoretical and practical contributions. Theoretically, it extends the Diffusion of Innovation Theory by examining how digital instructional leadership influences the integration of AI and pedagogy in K–12 schools. Additionally, the identification of AI integration as a mediating effect clarifies how leadership practices translate into innovative pedagogical actions. In parallel, incorporating trust in AI as a potential moderating variable enhances the theoretical framework by recognizing the ethical dimensions that influence teachers’ engagement with AI pedagogy, which have often been neglected in previous research on educational innovation. Practically, the study provides guidance for policymakers and school leaders on strengthening digital instructional leadership through targeted professional development that supports effective AI integration. This study also offers insights for teachers seeking to cultivate responsible, transparent, and ethically grounded uses of AI that align with their pedagogical aims. In sum, the integration of digital instructional leadership, AI integration, trust in AI, and AI pedagogy clarifies how school leaders can support AI-driven pedagogical transformation.

### Literature review and research hypotheses

This study draws on Diffusion of Innovations Theory [21] to examine the integration of AI in K–12 educational systems. Grounding the study in this framework highlights the role of school leaders in shaping the integration of AI among K–12 teachers.

### Diffusion of innovation theory

The Diffusion of Innovation Theory, developed by Everett Rogers, explains how new ideas, products, or technologies spread through a population over time [21, 22]. In a later expansion, Rogers [23] identified four key elements that influence the diffusion process: the innovation, communication channels, time, and the social system. The adoption process progresses through sequential stages, with individuals classified into five categories based on their propensity to embrace change: innovators, early adopters, early majority, late majority, and laggards [21]. First, innovators are individuals who introduce new ideas and are typically equipped with the financial resources and technical expertise to adopt emerging technologies. Second, *early adopters* are socially influential leaders who promote adoption through their credibility and visibility. Third, the *early majority* consists of a large and deliberate group that adopts innovations once their effectiveness has been established. Fourth, the *late majority* comprises more skeptical individuals who adopt an innovation only after it has gained widespread acceptance, often due to social pressure. Finally, *laggards* are the most resistant to change and tend to have minimal influence on the diffusion process.

The Diffusion of Innovation Theory has been extensively used to explain the adoption of new technologies in education [24–26]. In addition to describing diffusion patterns, the theory serves as an analytical lens for understanding how leadership, integration, pedagogy, and trust interact across the various stages of the diffusion process. Digital instructional leadership extends transformational and distributed leadership by incorporating the technology-specific expertise, such as knowledge of AI, that is necessary to enact the school organization’s vision for teaching and learning. Transformational leadership emphasizes shaping a shared vision and encouraging innovation through influence, stimulation, and individualized support, while distributed leadership highlights shared responsibility, collaboration, and the use of collective expertise across the school [27]. Moreover, frameworks such as the Technological Pedagogical Content Knowledge (TPACK help explain AI integration, which refers to teachers’ adoption and operational use of AI tools, from an AI pedagogy perspective, which involves the deliberate redesign of learning interactions [10, 28, 29]. However, when integrating AI, schools must also consider the sociocultural dimensions of learning, not only to enhance instructional tasks but also to provide opportunities for students to explore their identities, engage in dialogic reflection, and participate in emotionally meaningful learning experiences [30].

Digital instructional leadership reflects the initiation and persuasion stages of diffusion, as leaders acting as innovators or early adopters shape the vision, create supportive conditions, and model digital innovation to guide teachers’ adoption decisions [14, 20, 31]. AI integration consequently reflects the implementation stage, when early majority teachers begin embedding AI tools into instructional processes once they perceive observable benefits and institutional support [16, 32]. At a subsequent stage, AI pedagogy aligns with the confirmation stage of diffusion, where adoption evolves into sustained pedagogical transformation, signifying the internalization of AI as part of teaching practice [32]. Finally, trust in AI plays a psychological role in shaping the rate of adoption across the diffusion stages, as it influences the extent to which teachers are willing to incorporate digital tools into their daily instructional practice [17, 19, 33]. Accordingly, grounding these constructs in Rogers’ [21] Diffusion of Innovation Theory enables the study to identify the diffusion pathways through which leadership fosters innovation and to explain how affective and contextual variables, such as trust, shape the movement from adoption to pedagogical integration. This alignment enhances the explanatory power of the Diffusion of Innovation Theory in educational contexts by showing that the diffusion of AI pedagogy encompasses both the technical processes of adoption and the social dynamics that shape teachers’ engagement with AI [24, 32].

### Digital instructional leadership

Instructional leadership has emerged as a dominant paradigm in educational research and is widely recognized as a key determinant of school effectiveness [15, 34, 35]. Within this model, instructional leaders set and communicate the school’s academic goals, supervise instruction, monitor student progress, and create opportunities for teacher professional development [36]. They act as agents in shaping the curriculum, enhancing pedagogy, and cultivating a positive organizational climate to support student achievement [37]. Instructional leadership also emphasizes informed decision-making, enabling school leaders to evaluate the school’s culture and climate. This process is collaborative, involving principals, teachers, and other staff to ensure that decisions are well-informed, inclusive of diverse perspectives, and directed toward sustained school improvement [15, 38].

Building on the concept of instructional leadership, scholars have developed the concept of digital instructional leadership [14, 20, 31, 39]. This leadership style refers to a school leader’s capacity to use information technology effectively, understand and communicate the instructional demands, promote a clear vision for technology integration, and create meaningful learning opportunities that support all members of the school community in adopting and using digital tools [14, 20, 40]. Shepherd and Taylor [31] emphasize three interrelated domains: organizational change, vision, and professional development. Meanwhile, organizational change involves restructuring educational frameworks and operational processes to integrate digital tools into teaching and learning systems. In addition, vision involves a leader’s vision involves defining, communicating, and sustaining a clear direction for technology-enhanced education that aligns with broader institutional goals. Finally, professional development entails providing teachers with access to resources and support to build technological skills, confidence, and instructional strategies necessary for effective technology-mediated teaching.

Digital instructional leadership has attracted substantial scholarly interest during periods of major educational reform. During the COVID-19 period, scholarly attention intensified as school leaders were compelled to adopt this leadership approach in response to the rapid shift to digital learning environments [20, 39, 41]. In practice, this leadership approach supports both teachers and students by enabling effective technology integration that enhances teaching and learning. For example, Berkovic [41] found that principals’ digital instructional leadership indirectly influenced perceived student learning by enhancing teachers’ intrinsic motivation for digital instruction. Moulin and Soncin [39] also found that digital instructional leadership was linked to student outcomes. Moreover, Pietsch and Mah [14] found that digital instructional leadership has an influence on AI implementation in K–12 schools. Accordingly, we hypothesize:

- H1: Digital instructional leadership has a significant positive effect on AI Integration.
- H2: Digital instructional leadership has a significant positive effect on AI Pedagogy.

### The relationship between AI integration and AI pedagogy

In the twenty-first century, the integration of AI in K–12 education is accelerating as schools adopt AI technologies to support pedagogical innovation [32, 42]. Prior research suggests that integrating AI in K–12 education offers reciprocal benefits by enhancing teachers’ capacity for innovative instruction [43, 44] and enriching students’ learning experiences, particularly through active learning and increased engagement [45]. As this trend advances, there is growing interest in AI pedagogy, defined as the deliberate selection and application of AI tools to improve learning content, instructional delivery, and educational outcomes [10]. Kabudi et al. [3] emphasize that effective AI integration enhances pedagogical practices by enabling more personalized, engaging, and adaptive learning experiences. Similarly, Katona and Gyönyörű [8] argue that AI integration provides the technological foundation for innovative pedagogy. Meanwhile, a considerable body of literature indicates that AI-driven adaptive learning tools, in particular, support innovative teaching methods by providing real-time feedback that strengthens student motivation and engagement [44]. Thus, we hypothesize:

- H3: AI Integration has a significant positive effect on AI Pedagogy.

### AI integration as a mediator

AI integration has the potential to function as the key link between digital instructional leadership and AI pedagogy. When school leaders promote a shared digital vision, provide professional support, and encourage experimentation, they create an environment in which teachers feel confident using AI tools in their daily teaching [14]. Through this process, leadership influences how teachers adopt and integrate AI into their classroom practices [13], thus shaping their ability to design and deliver AI-based instruction. This mediating relationship suggests that the effect of leadership is realized not through direct influence on AI pedagogy but through the extent to which teachers integrate AI into instructional practice. Previous research also suggests that effective leadership often fosters pedagogical innovation indirectly, by shaping the organizational climate and enhancing teachers’ readiness for technology use [14, 16, 41]. In this context, AI integration serves as the practical means through which leadership promotes AI-driven teaching and learning. Accordingly, we hypothesize:

- H4: AI Integration mediates the relationship between Digital Instructional Leadership and AI Pedagogy.

### Trust in AI as a moderator

Trust in AI has been examined as a moderating variable in studies of AI use in educational contexts [17, 46]. Although trust is a key factor in AI adoption and acceptance [33], it can also reduce intention to use AI [17]. Research on AI acceptance consistently shows that trust is critical when educators adopt and integrate AI-driven tools into their teaching [18, 19]. Key constructs that shape trust in technology include reliability, functionality, helpfulness, performance, process, and purpose [33]. Ryan [47] argued that what is often described as ‘trust in AI’ is better understood as a form of reliance, since AI lacks the emotive states and moral agency required for genuine trust. Nevertheless, teachers who express greater trust in AI tend to incorporate these technologies more readily into their pedagogical practices. At the same time, although AI technologies offer significant benefits [48], they also present challenges that may influence teachers’ willingness to use them, including data privacy concerns Carmody et al. 2021 [49], algorithmic bias [9], and the risk of over-reliance on automated systems [50]. In particular, data privacy concerns highlight the ethical risks associated with collecting, storing, and using personal information, especially in technology-enhanced learning environments [50]. These concerns reinforce the importance of trust, and a prior study by Foroughi et al. [17] reported that trust in AI moderates the effects of perceived ease of use and perceived usefulness on attitudes toward A.

Chiu [46] noted that higher levels of trust will strengthen the positive impact of AI integration on AI pedagogy, as teachers with greater trust are more likely to perceive AI tools as reliable, thus engaging more deeply with innovative pedagogical practices. Conversely, lower trust may weaken this relationship, as skepticism or concerns about AI’s reliability and ethical implications could limit teachers’ willingness to fully embrace AI in their instructional practice [51]. Such an interpretation of trust’s role in AI adoption aligns with recent calls for more context-sensitive moderation analyses in educational technology research, which emphasize the importance of examining how individual and contextual factors shape technology adoption outcomes [18, 52]. These insights underscore trust in AI as an influential moderator in the relationship between AI integration and AI pedagogy. Therefore, we hypothesize:

- H5: Trust in AI moderates the relationship between AI integration and AI Pedagogy.

### Methodology

**Fig. 1:**
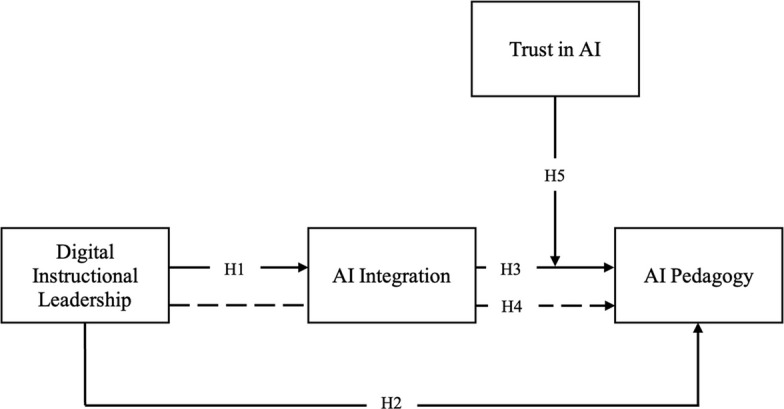
Conceptual model

This study employed a large-scale quantitative cross-sectional survey [53] to examine the hypothesized relationships among Digital Instructional Leadership, AI Integration, AI Pedagogy, and Trust in AI in K–12 education. The conceptual model is presented in Fig. 1.

### Participants and procedure

The study involved K–12 teachers in Taiwan, selected through purposive sampling to ensure representation across different school types and geographic regions (see Table 1). Purposive sampling is a non-probability sampling technique in which researchers deliberately select participants based on specific characteristics most relevant to the study’s purpose [54]. Participants were recruited through professional networks, and informed consent was obtained from all participants prior to data collection. After excluding 68 invalid responses, an intact sample of 842 valid responses was retained. To be eligible, participants were required to (1) be currently employed as a K–12 teacher and (2) have experience using AI tools in classroom instruction.

**Table 1:** Participant characteristics

Variable	Category	n	Percentage (%)
Age	< 30 years	118	13.90
	30–39 years	112	13.20
	40–49 years	224	26.30
	50–59 years	266	31.30
	> 60 years	122	14.30
Sex	Male	323	38.00
	Female	519	61.00
Educational qualification	Bachelor	244	28.70
	Master	562	66.00
	Doctorate	36	4.20
Teaching experience	< 1 year	104	12.20
	1–5 years	94	11.00
	6–10 years	125	14.70
	11–15 years	152	17.90
	> 15 years	367	43.10
School settings	Urban	592	69.60
	Suburban	171	20.11
	Rural	79	9.30
Teaching level	Elementary school	457	53.70
	Junior high school	241	28.30
	Senior high school	144	16.90
Total		842	100.00

### Instrument

The survey instrument comprised validated items adapted from prior research and measured on a five-point Likert scale ranging from 1 (strongly disagree) to 5 (strongly agree). Before distribution, the items were translated and reviewed by native Taiwanese experts, two of whom held degrees in English and had prior experience with back-translation procedures for survey instruments. In this study, psychometric equivalence tests were not performed because linguistic and conceptual equivalence can be adequately established through expert review, cognitive pretesting, and pilot testing, an approach recognized as valid in cross-cultural instrument adaptation Van de Vijver & Leung, 2011 [55, 56]. In addition to back-translation, we piloted the items with a group of in-service teachers and revised them until no respondent reported any ambiguity. Subsequently, all authors jointly reviewed the final version to ensure linguistic accuracy and appropriateness for the study context. The items are listed in the appendix.

- *Digital Instructional Leadership* was measured using four items adapted from Pietsch and Mah [14], assessing school leaders’ capacity to support digital and AI-enhanced teaching. The items capture how leaders promote pedagogical innovation and support teachers in integrating technology into instruction. The items were slightly modified to fit the goals of this study. A sample item is: “*Our principal helps teachers who have difficulties using digital media in their lessons.*”
- *AI Integration* was measured using three items adapted from Pietsch and Mah [14], focusing on the extent to which AI technologies are incorporated into instructional practices. The items reflect how teachers engage with AI tools to support teaching and learning in the classroom. A sample item is: *“We integrate AI-based tools (e.g., DeepL, ChatGPT) into our teaching.”*
- *Trust in AI* was assessed using four items adapted from Choi et al. [19], reflecting teachers’ perceptions of the reliability, fairness, and dependability of AI-based educational tools. The items capture the extent to which teachers view AI-generated decisions as trustworthy and aligned with ethical expectations in classroom contexts. A sample item is: *“I think that AI can provide a reliable decision.”*
- *AI Pedagogy* was assessed using four items adapted from Chiu et al. [10], focusing on teachers’ ability to apply AI tools with subject content and instructional strategies. The items reflect how teachers incorporate AI to design lessons, support personalized learning, and align technology with pedagogical objectives. A sample item is: *“I can choose an AI tool to use in my classroom that enhances what I teach, how I teach, and what students learn.”*

### Data analysis

We analyzed the data using SPSS 27 and SmartPLS 4.0. SPSS was used for descriptive statistics and Harman’s single-factor test to assess common method bias. For hypothesis testing, partial least squares structural equation modeling (PLS-SEM) was employed in SmartPLS, an approach particularly suited for predictive research [57]. We also examined mediation and moderation effects using bootstrapping with 5000 resamples and 95% confidence intervals (CIs), which provides stable parameter estimates and reliable CIs for indirect and interaction effects [58].

## Results

### Common Method Bias (CMB)

Two analytical approaches were conducted to assess common method bias (CMB). First, Harman’s single-factor test was conducted using principal component analysis. The results showed that the first unrotated factor accounted for 48.06% of the total variance, which is below the 50% threshold [59]. Second, we examined variance inflation factors (VIFs) as recommended by Kock and Lynn 2012 [60]. The VIF values for all predictor variables were below the recommended cutoff of 3.3, ranging from 1.000 to 1.258. Both tests indicate that CMB was not a significant issue in our study.

### Measurement model assessment

Confirmatory factor analysis (CFA) was conducted to examine the reliability, convergent validity, and discriminant validity of the measurement model [57]. Meanwhile, reliability was evaluated using Cronbach’s alpha and composite reliability (CR). As shown in Table 2, all constructs demonstrated strong internal consistency, with Cronbach’s alpha values ranging from 0.810 to 0.912, exceeding the recommended threshold of 0.70. Similarly, CR values ranged from 0.887 to 0.938, confirming good reliability. Next, convergent validity was assessed through standardized factor loadings and average variance extracted (AVE). All factor loadings exceeded 0.70, and all AVE values were above 0.50 [61], with estimates ranging from 0.701 to 0.792. Finally, discriminant validity was evaluated using the Fornell–Larcker criterion. As shown in Table 3, the square root of each construct’s AVE was greater than its correlations with any other construct, indicating satisfactory discriminant validity [61].

**Table 2:** Construct reliability and convergent validity

Construct	Item	Factor Loading	Cronbach's Alpha	CR	AVE
DIL	DIL1	0.899	0.912	0.938	0.792
	DIL2	0.902			
	DIL3	0.918			
	DIL4	0.839			
AII	AII1	0.830	0.810	0.887	0.724
	AII2	0.874			
	AII3	0.849			
AIP	AIP1	0.875	0.876	0.916	0.732
	AIP2	0.885			
	AIP3	0.891			
	AIP4	0.765			
TAI	TAI1	0.814	0.858	0.904	0.701
	TAI2	0.800			
	TAI3	0.864			
	TAI4	0.869			

*DIL*, Digital Instructional Leadership, *AII* AI Integration, *AIP* AI Pedagogy, *TAI* Trust in AI

**Table 3:** Descriptive statistics and discriminant validity

Construct	M	SD	Skewness	Kurtosis	AII	AIP	DIL	TAI
AII	3.745	0.971	−0.418	0.101	**0.851**			
AIP	3.865	0.863	−0.551	0.974	0.774	**0.856**		
DIL	3.598	0.772	−0.661	−0.031	0.264	0.238	**0.890**	
TAI	3.551	0.705	−0.406	0.256	0.403	0.418	0.198	**0.838**

The bold diagonal values represent the square root of the AVE and are greater than the correlations with all other constructs

### Structural model assessment

**Fig. 2:**
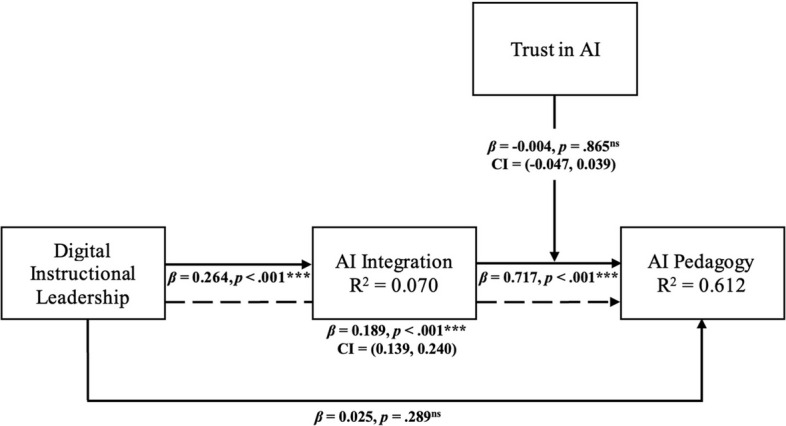
Structural model results. *Note.* 1. Solid lines indicate direct effects, whereas the dashed line indicates the indirect path. 2. ***p <.001. 3.* ns* indicates not supported

To empirically test the hypothesized relationships, we assessed key indicators, including the coefficient of determination (R2), predictive relevance (Q2), effect sizes (f2), and the statistical significance of path coefficients [57]. R2 represents the proportion of variance in endogenous constructs explained by their exogenous predictors and provides an estimate of the model’s explanatory power. As shown in Fig. 2, the model explained 61.2% of the variance in AI Pedagogy (R2 = 0.612), indicating strong explanatory power, and 7.0% of the variance in AI Integration (R2 = 0.070), reflecting a modest level of explained variance. In addition, the model demonstrated predictive relevance, with Q2 values of 0.129 for AI Pedagogy and 0.066 for AI Integration, both of which exceeded zero [57]. While both values indicate predictive relevance, the higher Q2 for AI Pedagogy suggests stronger predictive capability for this construct compared to AI Integration. Overall, the model demonstrates satisfactory explanatory power, particularly for AI Pedagogy.

The results revealed that digital instructional leadership had a significant positive effect on AI integration (*β* = 0.264, *t* = 7.75, *p* < 0.001), supporting H1. The effect size (*f*2 = 0.075)indicated a small to moderate practical impact. However, digital instructional leadership did not directly predict AI pedagogy (*β* = 0.025, *t* = 1.06, *p* = 0.289, *f*2 = 0.001), and H2 was therefore not supported. By contrast, AI integration showed a strong and statistically significant positive effect on AI pedagogy (*β* = 0.717, *t* = 32.37, *p* < 0.001), with a large effect size (*f*2 = 1.053), providing strong support for H3. In addition, mediation analysis confirmed a significant indirect effect of digital instructional leadership on AI pedagogy through AI integration (β = 0.189, *t* = 7.44, *p* < 0.001, 95% CI [0.139, 0.240]), supporting H4. These findings suggest that AI integration fully mediates the relationship between digital instructional leadership and AI pedagogy. Finally, the moderating effect of trust in AI on the relationship between AI integration and AI pedagogy was not statistically significant (β = − 0.004, *t* = 0.17, *p* = 0.865, 95% CI [− 0.047, 0.039]), and H5 was not supported. The detailed descriptions are provided in Table 4.

**Table 4:** Hypothesis test results

Hypothesis	Relationships	β	t-value	p-value	f^2^	VIF	Decision
*Direct effect*							
H1	DIL → AII	0.264	7.746	0.000	0.075	1.000	Supported
H2	DIL → AIP	0.025	1.060	0.289	0.001	1.087	Not supported
H3	AII → AIP	0.717	32.371	0.000	1.053	1.258	Supported
*Indirect effect*							
H4	DIL → AII → AIP	0.189	7.436	0.000	—	—	Supported
*Moderation effect*							
H5	TAI x AII → AIP	−0.004	0.171	0.865	—	—	Not supported

## Discussion and conclusion

### Does principals’ digital instructional leadership influence AI integration and AI pedagogy among K–12 teachers? (RQ1)

Our first research question investigated whether digital instructional leadership impacts the integration of AI and AI pedagogy in K–12 schools. The results indicated that digital instructional leadership significantly predicted AI integration (H1). Although digital instructional leadership significantly predicted AI integration, the relatively modest coefficient of determination for this construct suggests that additional factors not included in the model may also play an important role. Prior research indicates that elements such as institutional support, teacher digital literacy, technological infrastructure, and school-level resource availability often shape teachers’ willingness and capacity to adopt AI tools [51, 62]. While these findings align with Taiwan’s current digital leadership policy which prioritizes infrastructure and teacher training [63, 64], implementation is still ongoing and various obstacles might arise as schools adopt AI. Consistent with Pietsch and Mah [14] who reported similar patterns in Germany, our result likewise shows a positive effect of digital instructional leadership on AI integration in Taiwan. This underscores the need for school leaders to develop expertise in emerging instructional technologies, particularly AI integration in 21st-century learning environments. In parallel, school leaders serve as role models by supporting targeted professional development initiatives, exercising innovative and transformational leadership practices, and prioritizing curriculum redesign [31]. This pattern aligns with Diffusion of Innovation Theory [21], which emphasizes the role of leaders in facilitating the initial uptake of innovation within K–12 schools. In practical terms, a clear vision from school leaders for AI use equips teachers to integrate AI tools with greater confidence [16, 65].

Surprisingly, no significant correlation was found between digital instructional leadership and AI pedagogy (H2). This nonsignificant result may not signal a lack of influence; rather, it likely reflects the multifaceted and evolving nature of both constructs and the need for closer alignment among leadership strategies, professional development, ethical considerations, and pedagogical innovation. The nonsignificant direct path from digital instructional leadership to AI pedagogy can also be understood in light of research showing that its influence often operates indirectly through teacher motivation and professional learning communities (PLCs). Although digital instructional leadership can motivate teachers by offering access to digital resources [41], the extent to which these efforts manifest into classroom practice is frequently shaped by PLCs. This is because PLCs may affect teacher’s innovation to adopt technology which also affected by transformational leadership [66].

For many teachers, AI pedagogy is still relatively new [10]. Even when school leaders promote AI integration, the specific requirements, including curriculum guidelines, may not be fully understood. This finding echoes Wang and Cheng’s [67] observation that teachers often face internal barriers, including negative attitudes toward AI, incomplete or inaccurate understandings of its capabilities, misconceptions about its use, and low confidence in applying it in teaching. Additional challenges include uncertainty about hardware investments, time constraints, ethical concerns, and limited knowledge of AI’s pedagogical applications [13]. The age distribution of participants may also have contributed to the result. Consistent with this possibility, Bakhadirov et al. [62] reported that younger teachers typically exhibit higher acceptance and use of the technology than senior teachers. Given that many participants in our sample were senior teachers, they may have been more reluctant to integrate AI into pedagogical practice. In addition, although digital instructional leadership can articulate a vision for innovation, substantive changes in classroom practice require sustained professional development. In this regard, effective AI pedagogy in K–12 schools depends on targeted professional development implemented with support from school leaders [7, 10, 52].

Our findings further show that AI integration significantly predicts AI pedagogy (H3), reinforcing prior evidence that integrating AI in K–12 education enhances teachers’ capacity for innovative instruction [43, 44]. Consistent with Katona and Gyönyörű [8], AI-supported practices can yield innovative teaching models that blend human judgment with AI, moving pedagogy in new directions. The TPACK framework, a widely recognized model of teacher digital competence used in professional development initiatives, helps explain this relationship [10, 28, 29]. Within this framework, AI is viewed as a digital technology that enhances the interplay among technological, pedagogical, and content knowledge [29], This integration benefits both teachers and students by advancing innovative pedagogy that builds student autonomy, supports differentiated instruction, and promotes collaborative learning Chounta et al. 2023 [5, 8, 68].

Additionally, the mediation analysis revealed that AI integration mediated the relationship between digital instructional leadership and AI pedagogy (H4). This indicates that AI integration operates as the mechanism through which digital instructional leadership influences AI pedagogy. In other words, it serves as a critical link that turns leadership into the adoption of AI-informed pedagogical practice. From a broader perspective, digital instructional leadership entails the intentional use of AI by school leaders to support and improve teachers’ instructional practices. It includes leadership practices that use AI tools to guide the implementation of new technology trends and improve instructional outcomes. Recent studies have highlighted that digital instructional leadership plays a pivotal role in shaping teachers’ competence in adopting emerging technologies [14, 41]. Through targeted professional development, school leaders support teachers in building the capacity to integrate AI meaningfully into classroom instruction. Our findings align with existing literature suggesting that the influence of leadership on instructional practice is often indirect, mediated by key enablers such as teacher beliefs, digital competence, and ICT integration. For instance, Schmitz et al. [69] found that school leadership influences teachers’ use of technology through the mediating roles of teacher beliefs, digital skills, and digital infrastructure. Similarly, Vermeulen et al. [70] reported that ICT integration plays a crucial mediating role in the relationship between principal leadership and pedagogical practices. Accordingly, sustained guidance from skilled digital instructional leaders is essential to ensure alignment with instructional goals, and the thoughtful integration of AI remains vital for enhancing the quality of teaching and learning [40].

### Does teachers’ trust in AI moderate the relationship between AI integration and AI pedagogy? (RQ2)

In response to RQ2, we tested whether trust in AI moderated the relationship between AI integration and AI pedagogy (H5). Although prior research reports that trust in AI can moderate intention to use AI [17], our results, interestingly, showed no significant moderating effect. Our findings are consistent with previous research, which also reported an unexpected nonsignificant moderating effect of trust in AI on the relationship between explainability and user awareness [46]. This result may be attributable to several theoretical and contextual factors. One plausible explanation is limited variability in trust across our sample; when trust levels are uniformly high or low, AI integration may exert a stronger influence on pedagogical practice. Another possibility is that other factors, such as AI literacy, habitual reliance on AI, or perceived usefulness, play a more decisive role in shaping pedagogy [42, 52].

In the context of Taiwanese schools, the absence of a moderating effect of trust in AI may reflect broader cultural and institutional norms concerning digital trust. In this study, trust in technology may be shaped by collective experiences, institutional legitimacy, and policy-driven adoption rather than individual perceptions [71]. Teachers may place greater emphasis on institutional guidance and policy mandates than on personal trust, especially when AI adoption is embedded within national curriculum reforms and school-level directives. This tendency is particularly pronounced within Taiwan’s K–12 system, where the implementation of AI is structured in accordance with the 12-Year Basic Education Curriculum Guidelines, which place strong emphasis on digital literacy and the cultivation of adaptive learning competencies [72]. Under such educational conditions, teachers are more likely to employ AI tools as supports for differentiated instruction rather than as autonomous decision-making technologies. Consequently, their pedagogical use of AI may be shaped more by institutional mandates than by trust in the technology [8, 19]. These findings suggest that cultural and contextual norms in Taiwanese schools may shape how trust in AI is operationalized and experienced, potentially diminishing its moderating role in pedagogical practice. Trust in AI, in this context, appears to be guided more by institutional expectations than by teachers’ individual evaluative judgments.

A broader insight emerging from the findings of our study is that trust in AI may operate differently in educational systems where technology use is closely governed by institutional policy. In highly structured environments, teachers often view AI as an extension of established directives rather than as a tool that requires personal evaluative judgment. As a result, the significance of teachers’ trust may diminish when pedagogical decisions are already guided by curriculum standards and administrative expectations. This dynamic shows that the influence of trust in AI depends, in part, on the level of autonomy teachers hold in adopting and experimenting with digital tools. Viewed more broadly, the place of trust in AI can be interpreted as dependent on the institutional arrangements that frame teachers’ pedagogical judgments.

### Implications

#### Theoretical implications

It is noteworthy that this study advances understanding of how digital instructional leadership shapes AI pedagogy through the mediating role of AI integration among K–12 teachers. Grounded in the Diffusion of Innovation Theory, the findings indicate that digital instructional leadership does not exert a direct influence on AI pedagogy; rather, its impact is mediated by the adoption and integration of AI tools into teaching practice. Furthermore, when school leaders act as early adopters, their visible use of AI promotes adoption among the early majority (teachers). This extends prior work by clarifying that leadership’s influence on pedagogical innovation emerges once technological integration becomes embedded in instructional routines [14, 73]. In addition, this study enriches the literature by documenting a nonsignificant moderating role of trust in AI, suggesting that system-level directives and institutional supports may overshadow teachers’ trust when pedagogy is being shaped.

#### Practical implications

Two pedagogical implications arise from this study. First, for policymakers and school leaders, the findings highlight the importance of prioritizing AI integration as a foundation for pedagogical transformation. Sustained professional development should extend beyond technical training to strategically embed AI in curriculum planning and instructional design [13, 52]. Education authorities should strengthen digital instructional leadership by investing in digital infrastructure, issuing clear implementation guidelines, and providing ongoing leadership development on the use of AI in education. Accordingly, school leaders can further guide teachers in refining AI pedagogy through sustained professional development. That is, digital instructional leadership practices should emphasize modeling effective AI use and cultivating a supportive culture for AI pedagogy. Second, for teachers, the findings offer guidance on strengthening AI pedagogical knowledge, which can advance innovative teaching practices and enhance student learning. To achieve this goal, teachers need to build AI competency by developing the knowledge, skills, and professional values essential for effective instruction in the age of AI [7]. In practice, this involves selecting appropriate AI tools and applying them effectively to strengthen AI pedagogy [10].

### Limitations and future research directions

Although this study has yielded findings that offer both theoretical and pedagogical implications, its design is not without flaws. First, since our study employs a cross-sectional design, it limits causal inferences regarding the relationships among variables. Future studies are needed to capture the dynamic nature of AI adoption and to assess how leadership influences evolve as implementation matures. Second, the reliance on self-reported measures may also introduce bias. Therefore, future research should consider incorporating multiple data sources, such as observations and interviews, to enhance the validity and reliability of the findings. Third, the study employed purposive sampling of teachers who were already using AI tools, which raises concerns about representativeness and may limit the generalizability of the findings. Future studies could adopt broader or probabilistic sampling strategies to include teachers with varying levels of experience with AI. Fourth, this study was conducted in Taiwan’s K–12 schools, which may limit the generalizability of the findings to other countries. We are hopeful that future studies could explore variations across educational systems and geographic locations. Fifth, given the nonsignificant moderating effect of trust in AI on the relationship between AI integration and AI pedagogy, this relationship warrants more rigorous examination. Ultimately, future research could investigate how various leadership models, including transformational, distributed, and instructional leadership, influence AI-based pedagogical innovation in diverse school settings.

## Data Availability

The datasets used and/or analyzed during the current study are available from the corresponding author on reasonable request.

## Appendix

### Measurement items

Digital Instructional Leadership (adapted from [14])

1. Our principal helps teachers who have difficulties using digital media in their lessons.
2. The principal addresses new teaching methods with digital media in schools.
3. The principal acts as a role model for other teachers through their own use of digital media in teaching.
4. The principal regularly visits lessons in which digital media are used.

AI Integration (adapted from [14])

1. We integrate AI-based tools (e.g., DeepL, ChatGPT) into our teaching.
2. We regularly use AI-powered applications (e.g., DeepL, ChatGPT).
3. We are exploring the topic of AI and have launched our first projects.

AI Pedagogy (adapted from [46])

1. I can choose an AI tool to use in my classroom that enhances what I teach, how I teach, and what students learn.
2. I can choose an AI tool that enhances my teaching subject content for a lesson.
3. I can teach lessons that appropriately combine my teaching subject, AI tools, and teaching approaches.
4. I can help others coordinate the use of subject content, AI tools, and teaching approaches.

Trust in AI (adapted from [19])

1. I think that AI can provide a reliable decision.
2. I think that decisions from AI are fair.
3. I feel that AI is dependable.
4. Overall, I can trust AI.
